# Labeling and analysis of chicken taste buds using molecular markers in oral epithelial sheets

**DOI:** 10.1038/srep37247

**Published:** 2016-11-17

**Authors:** Prasangi Rajapaksha, Zhonghou Wang, Nandakumar Venkatesan, Kayvan F. Tehrani, Jason Payne, Raymond L. Swetenburg, Fuminori Kawabata, Shoji Tabata, Luke J. Mortensen, Steven L. Stice, Robert Beckstead, Hong-Xiang Liu

**Affiliations:** 1 Regenerative Bioscience Center, Department of Animal and Dairy Science, College of Agricultural and Environmental Sciences, University of Georgia, Athens, GA, USA; 2 Department of Poultry Sciences, College of Agricultural and Environmental Sciences, University of Georgia, Athens, GA, USA; 3 Laboratory of Functional Anatomy, Faculty of Agriculture, Kyushu University, Fukuoka, Japan; 4 College of Engineering, University of Georgia, Athens, GA, USA

## Abstract

In chickens, the sensory organs for taste are the taste buds in the oral cavity, of which there are ~240–360 in total number as estimated by scanning electron microscopy (SEM). There is not an easy way to visualize all taste buds in chickens. Here, we report a highly efficient method for labeling chicken taste buds in oral epithelial sheets using the molecular markers Vimentin and α-Gustducin. Immediate tissue fixation following incubation with sub-epithelially injected proteases enabled us to peel off whole epithelial sheets, leaving the shape and integrity of the tissue intact. In the peeled epithelial sheets, taste buds labeled with antibodies against Vimentin and α-Gustducin were easily identified and counted under a light microscope and many more taste buds, patterned in rosette-like clusters, were found than previously reported with SEM. Broiler-type, female-line males have more taste buds than other groups and continue to increase the number of taste buds over stages after hatch. In addition to ovoid-shaped taste buds, big tube-shaped taste buds were observed in the chicken using 2-photon microscopy. Our protocol for labeling taste buds with molecular markers will factilitate future mechanistic studies on the development of chicken taste buds in association with their feeding behaviors.

In chickens, chemical signals in feed are transduced by taste buds, the taste sensory organs, and transmitted to the brain as neural information that motivates and guides feed intake. Chicken taste buds are different from those of mammals in several aspects. For example, chicken taste buds are ovoid-shaped and they appear mostly near the openings of the salivary glands. Unlike mammals, in which taste buds are primarily located in the tongue, only 2% of chicken taste buds are located in the posterior region of the tongue. The majority of chicken taste buds are located in the epithelium of the palate (69%) and in the base of the oral cavity (anterior mandibular gland region, 29%)^1^,^2^,^3^.

The number of taste buds correlates positively with taste sensitivity in chickens. A greater number of taste buds results in higher sensitivity to bitter taste^4^. It has been reported that chicken taste buds develop at late embryonic (E) stages, and that the number of taste buds remains constant after E19^5^. The average number of taste buds varies widely and falls into a range of 240–360, depending on the breed and gender of the chicken (for example, broiler-type males have more taste buds than males in the layer-type)^2^,^3^.

Previously, the distribution and number of chicken taste buds was determined by counting taste pores using scanning electron microscopy (SEM)^2^,^3^, which only allows identification of mature taste buds with a taste pore open to the surface of oral epithelium^2^,^5^. An appropriate method for visualizing all chicken taste buds in order to observe their distribution pattern and acquire accurate counts has not existed. Whole mount tissue, commonly used to observe organs and tissue of interest, may be used to study taste buds in chicken. We recently developed a simple method for labeling and quantifying mouse taste buds in which taste cells in intact tongue epithelial sheet were labeled by molecular markers^6^. This protocol, if applied to chicken gustatory tissue, will facilitate research on a possible change in the number of taste buds present during development. The change may be associated with taste sensitivity and thus the feeding behavior of chickens.

In chickens, molecular markers have been used to label taste buds in tissue sections^7^,^8^,^9^. For instance, previous studies have demonstrated that Vimentin and α-Gustducin each label a subpopulation of taste buds in chicken^8^,^9^. Our recent data showed that Vimentin and α-Gustducin labeled largely overlapping populations of taste bud cells^10^. Each molecular marker labeled only a subpopulation of taste bud cells in post-hatch chickens, however, both markers were observed in all the taste buds examined. Thus, α-Gustducin and Vimentin are potentially useful molecular markers for visualizing chicken taste buds in whole mount tissue.

In the present study we tested a protocol for peeling off intact oral epithelial sheets in post-hatch broiler-type chickens. Taste bud labeling with the molecular markers Vimentin and α-Gustducin was successful in peeled epithelial sheets from the base of the oral cavity, the palate, and the posterior region of the tongue, areas where taste buds are located. We found that antibodies to molecular markers present in the taste buds labeled many more taste buds than previously counted using conventional SEM methods. The number of taste buds was higher in female-line males than other groups and continued to increase after hatch, providing a temporal window for mechanistic studies and for potentially regulating taste bud development and nutrient sensing. Moreover, in the peeled tissue, 2-photon microscopy was successfully used to examine the 3-D structure of chicken taste buds. Our methods are useful for future studies on chicken gustatory function, which is essential for making nutritive choices under motivation to intake feed. Also, the protocol may have broad application across fields, i.e., it is applicable to all epithelial appendages in different species.

## Results

### Oral epithelial sheet peeling and taste bud labeling

**Figure 1:**
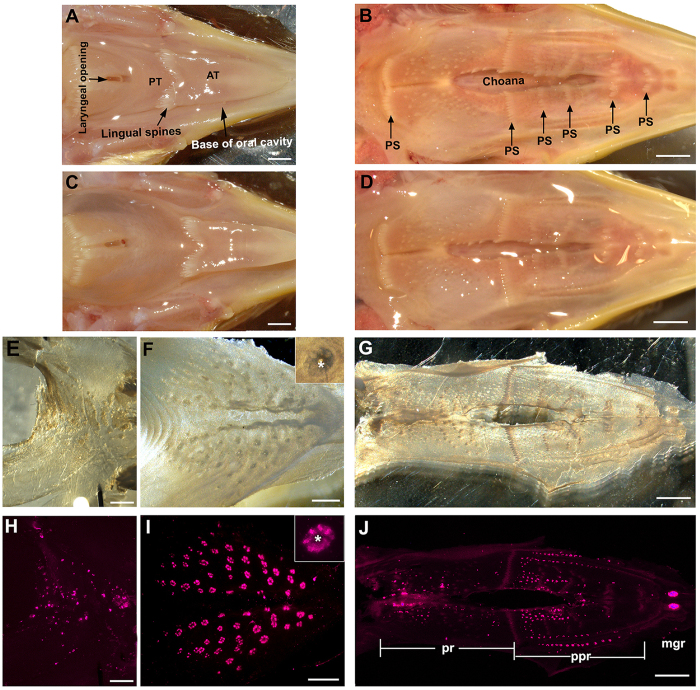
Representative photomicrographs of the tongue, base of the oral cavity and palate in P3 chickens to illustrate the major steps in the protocol for taste bud labeling in peeled oral epithelial sheets. (**A**) A lower beak to show the base of the oral cavity dissected with the tongue *in situ*. AT: anterior tongue; PT: posterior tongue. (**B**) An upper beak to show the structure of the palate. PS: palatal spines. (**C,D**) Swollen tissue of the base of the oral cavity and posterior tongue (**C**), and the palate (**D**) after the sub-epithelial injection of proteases, which is less obvious in the palate. (**E–G**) Bright-field images of epithelial sheets from the posterior tongue (**E**), the base of the oral cavity (**F**), and the palate (**G**). (**H–J**) Photomicrographs of epithelial sheets taken under a fluorescence stereomicroscope after immunoreaction against Vimentin. Purple signals show the immunosignals of Vimentin in taste buds. mgr: maxillary gland region; ppr: palatine papillae region; pr: posterior region. Insets in (**F**) and (**I**) illustrate a cluster of taste buds surrounding a salivary gland opening (asterisks). Scale bars: 2 mm for (**A–D)** and (**G,J**); 1 mm for (**E,F)** and (**H,I**).

To efficiently label all taste buds, oral epithelial sheets from the palate, the base of the oral cavity, and the posterior region of the tongue in post-hatch chickens were peeled using our recently reported method for mouse tongue, with some modifications^6^. The major steps are shown in Fig. 1. In brief, dissected lower (Fig. 1A) and upper (Fig. 1B) beaks were given a sub-epithelial injection of proteinase into the oral tissue (Fig. 1C,D). Following incubation with the injected proteinase for 2 hr at 37 °C, immediate fixation of the tissue in 4% PFA for 1 hr at room temperature helped to maintain the shape and integrity of the epithelial sheets (Fig. 1E,G). Salivary gland openings (asterisk, Fig. 1F inset) in the peeled epithelium were seen as holes under a stereomicroscope and were surrounded by dark structures where taste buds are primarily located (Fig. 1F).

To better preserve the tissue for the long immunoreaction process, the peeled epithelial sheets were further fixed in 4% PFA for 2 hr and then the viability of taste bud labeling was tested using an immunoreaction against Vimentin. Clustered structures, some of which surrounded salivary gland openings, were labeled with Vimentin immunosignals. Vimentin immunosignals were distributed in the posterior tongue (Fig. 1H), anterior mandibular region of the base of the oral cavity (Fig. 1I), and the palate (Fig. 1J). In addition, labeled taste buds were seen in extending to the edge of the base of the oral cavity. Individual buds labeled by Vimentin immunosignals were easily identified and were distributed in clusters in a rosette-like pattern, more uniform and typical in the base of the oral cavity (Fig. 1I inset).

**Figure 2:**
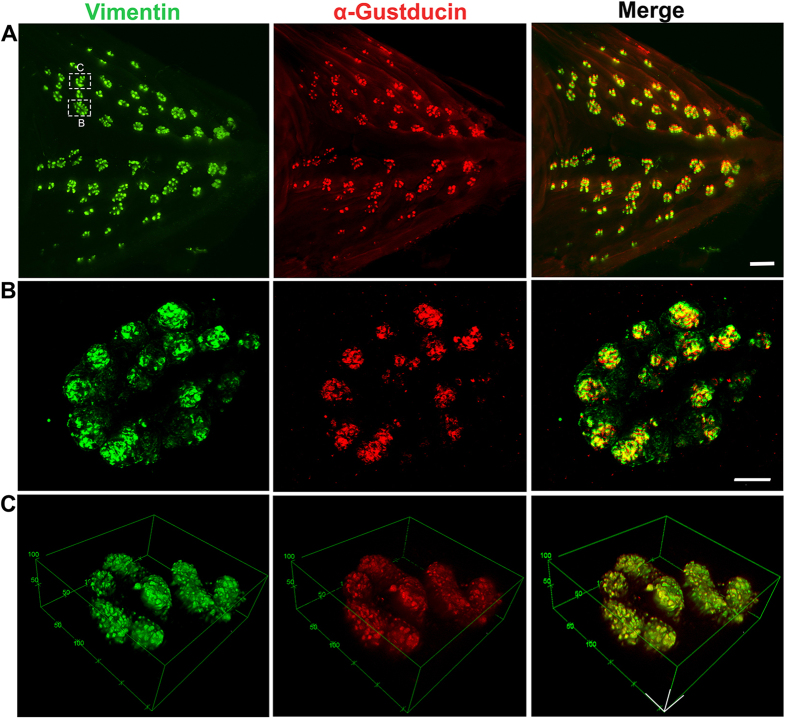
Photomicrographs illustrate the distribution of Vimentin (green) and α-Gustducin (red) immunoreactivity in taste buds in an epithelial sheet from the base of the oral cavity in a P3 male chicken. (**A**) Photomicrographs taken under a fluorescent stereomicroscope show the overlapping distribution of Vimentin and α-Gustducin immunosignals in taste buds. (**B**) Laser-scanning confocal photomicrographs (Z projection) were taken at high magnification to show individual taste buds in a cluster. Vimentin and α-Gustducin signals were seen in all taste buds and largely overlapped in the taste bud cells. Taste buds were arranged in a rosette pattern. (**C**) 3-D images taken under a 2-photon microscope illustrate the separated individual and ovoid-shaped buds surrounding a salivary gland opening. Scale bar: 500 μm in (**A**), 25 μm in (**B**), 50 μm in (**C**).

**Figure 3:**
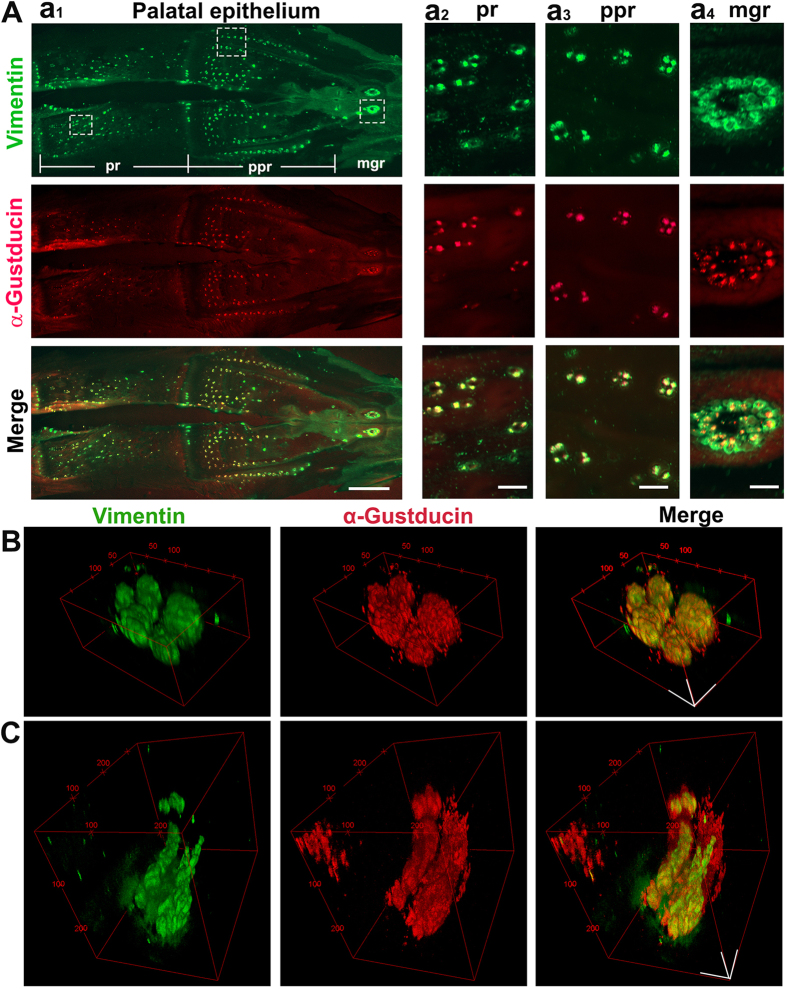
Taste buds in the palate. (**A**) Photomicrographs of a palatal epithelial sheet from a P3 female chicken labeled with Vimentin (green) and α-Gustducin (red) to illustrate the distribution pattern of taste buds. Vimentin and α-Gustducin immunosignals were overlapping in taste buds. Squares with dashed lines mark the areas for the higher magnification images in a_2–4_ columns in the maxillary gland region (mgr), palatine papillae region (ppr) and posterior region (pr) of the palate, respectively. Scale bars: 2 mm for a_1_, 200 μm for a_2–4_. (**B,C)** 2 photon 3-D reconstructed images of a taste bud cluster showing ovoid-shaped (**B**) and tube-shaped (**C**) taste buds in the palatine papillae region and posterior region. Scale bars: 50 μm in (**B**) and (**C**).

Peeling the palate epithelial sheets was more challenging, likely due to the smaller sub-epithelial space, lower holding capacity of the protease solution and abundant protruding palatine spines (arrows, Fig. 1B). After the enzyme injection, swollen palatal tissue was less obvious (Fig. 1D) compared to the posterior tongue and base of the oral cavity (Fig. 1C). Nonetheless, it was still possible to obtain intact epithelial sheets (Fig. 1G, brightfield) for taste bud labeling with Vimentin (Fig. 1J). In the palate epithelial sheets, taste buds labeled with Vimentin were distributed in a unique pattern (Fig. 1J). Similar to previous reports^2^,^3^, Vimentin immunosignals were mainly located in three tissue regions: (1) anterior to the lateral palatine wrinkles (maxillary gland region) (mgr, Fig. 1J), (2) lateral to the nasopalatal region (palatine papilla region) (ppr, Fig. 1J) and (3) in a posterior region adjacent to the choanal opening (pr, Fig. 1J). For further analyses, we focused on the base of the oral cavity and palate where taste buds are primarily located.

### Distribution and structure of taste buds labeled with Vimentin and α-Gustducin in the base of the oral cavity and palate

It has been reported that α-Gustducin is specifically expressed in chicken taste buds^9^,^11^. Two molecular markers, Vimentin and α-Gustducin, were used to label taste buds in the epithelial sheets of the base of the oral cavity in P0 to P8 chickens. The distribution and structure of the labeled taste buds were analyzed at both the organ and cellular levels (Fig. 2). Vimentin and α-Gustducin immunosignals were present in the same population of taste buds in the epithelial sheets (Fig. 2A). To confirm whether the fluorescent “dots” seen under the stereomicroscope were individual taste buds and could be used for further quantitative analysis, Vimentin and α-Gustducin labelling was further verified by laser-scanning confocal (Fig. 2B) and 2-photon (Fig. 2C) microscopy. The Z-projection of laser-scanning confocal photomicrographs and 3-D reconstructed 2-photon images of a cluster of taste buds from an epithelial sheet illustrated a bud cluster with a rosette-like arrangement of individual taste buds that were distinct from each other (Fig. 2B,C). The number of taste buds obtained from the Z-projection and 3-D images was identical to that obtained from images taken under a light stereomicroscope. Vimentin and α-Gustducin immunosignals each labeled a significant yet overlapping subpopulation of taste bud cells and every detected taste bud contained label. The 3-D images also show the egg-shaped structure of individual taste buds (Fig. 2C).

As mentioned above, peeling the palate epithelial sheets was more challenging. The taste bud labeling at selected stages (P0 and P3) using two molecular markers, Vimentin and α-Gustducin, was performed in the epithelial sheets of the palate. Similar to the labeling in the base of the oral cavity, Vimentin and α-Gustducin immunosignals were present in the same population of taste buds in the palatal epithelial sheets (Fig. 3A). In the maxillary gland opening region of the palate, two large clusters of taste buds were brightly labeled, each comprised of multiple taste buds (mgr, Fig. 3A). In the palatine papilla region, two lines of taste bud clusters were observed on both lateral sides, and a scattered distribution of taste buds was observed in the medial areas (ppr, Fig. 3A). Scattered taste buds were observed in the posterior palate (pr, Fig. 3A). Additionally, Vimentin and α-Gustducin labelling was further verified by 2-photon (Fig. 3B,C) microscopy. 2-photon 3-D reconstructed images of a taste bud cluster showed ovoid-shaped buds forming a rosette pattern in the palatine papilla region (Fig. 3B), similar to that seen in the base of the oral cavity, and elongated tube-shaped buds in the posterior region (Fig. 3C). Taste bud structure varied within the palate epithelium.

### Quantitative analysis of Vimentin labeled taste buds in the base of the oral cavity and palate

**Figure 4:**
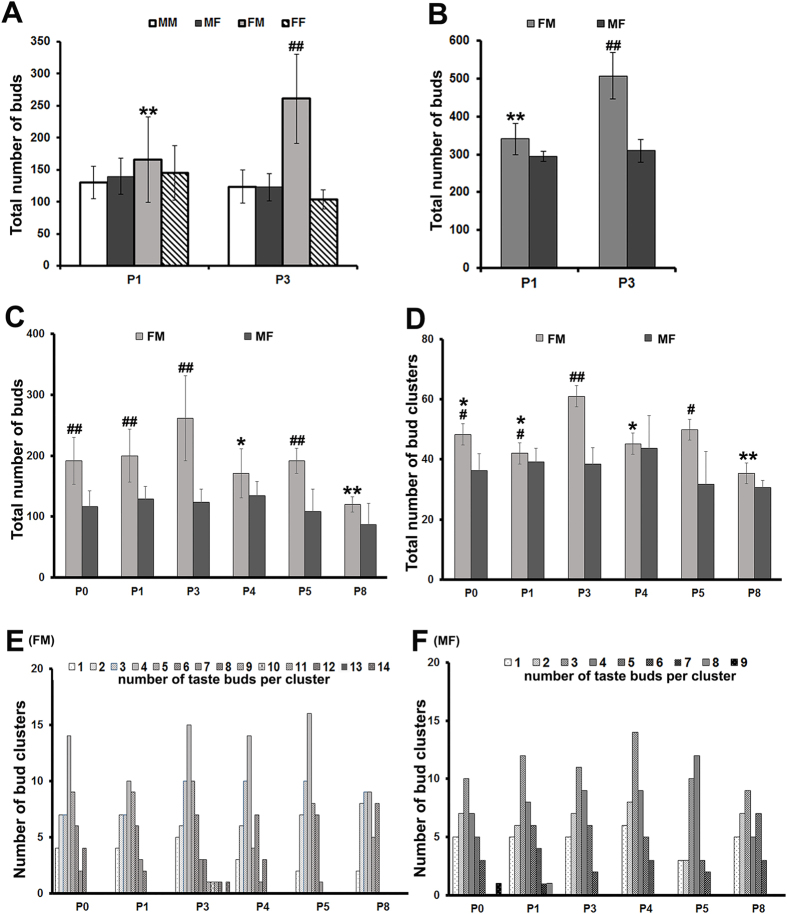
Quantitative analysis of total taste bud number (**A–C**), total number of bud clusters (**D**), and number of taste buds per cluster (**E,F**) in chickens at selected stages (P0–8). The data were obtained from epithelial sheets of the base of the oral cavity (**A,B**,**D–F**) and palate (**C**) immunoreacted against Vimentin. (**A,B**) Histograms represent the average (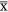 ± SD, n = 3–7) total individual taste bud numbers in male-line males (MM), male-line females (MF), female-line males (FM) and female-line females (FF) in the base of the oral cavity (**A**) and taste bud numbers in FM and MF chickens in the palate at P1 and P3 (**B**). (**C,D**) Histograms represent the average (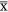 ± SD, n = 3–7) total numbers of individual taste buds (**C**) and bud clusters (**D**) in FM and MF chickens at P0–P8. (**E,F**) The histograms show variation in taste bud number within a taste bud cluster (1–14 in FM and 1–9 in MF), illustrating most abundant cluster sizes in males (i.e., 4 taste buds in a cluster) (**E**) and females (i.e., 3 taste buds in a cluster) (**F**). ^*#*^*P* ≤ *0.05*,^*##*^*P* ≤ *0.01* compared to other group(s) at the same stage, *P ≤ *0.05*, **P ≤ *0.01* compared to P3 male chicken group.

The total numbers of individual taste buds were quantified using Vimentin immunosignals in the base of the oral cavity of both males and females from both male- and female-line at P1 and P3 (Fig. 4A). There was no significant differences in the number of total taste buds at P1 among the groups of the broiler-type chickens (one-way ANOVA, F(3,23) = 0.71, *P* = 0.558). However, at P3 there was a significant difference among the groups (one-way ANOVA, F(3,23) = 23.56, *P* < 0.01). The P3 female-line males had a significantly higher number of taste buds compared to the other groups at P3 and all four groups at P1 (*P* < 0.01, Post-hoc Tukey HSD following two-way ANOVA). For the further analyses of taste bud numbers in the palate at selected stages (P1 and P3) (Fig. 4B) and in the base of the oral cavity across a broader range of stages (P0–P8), female-line male and male-line female chickens (Fig. 4C–F) were used.

In the palate (Fig. 4B), using Vimentin immunosignals to quantify the total number of taste buds in the female-line male and male-line female chickens at P1 and P3, we found that the total number of taste buds was higher in P3 males than females (*t*-test, *P* < 0.01). In addition, the P3 males had an increased number of taste buds compared to P1 males (*t*-test, *P* < 0.01). The total number of taste buds with Vimentin immunosignals in the palate epithelial sheets averaged 588 ± 19 (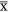 ± SD) at P3 (n = 3).

In the base of the oral cavity of chickens at a broad range of stage (P0–P8) (Fig. 4C), the overall total number of taste buds in female-line males was statistically higher than that in male-line females (Fig. 4C, two-way ANOVA, (F (5,49) = 2.61, *P* < 0.05). *t*-tests showed that the number of taste buds in the base of the oral cavity of female-line male chickens was higher than that in male-line females at P0 (*P* < 0.01), P1 (*P* < 0.01), P3 (*P* < 0.01), and P5 (*P* < 0.01). Among post-hatch stages, the differences were statistically significant in males (one-way ANOVA, F(5,28) = 4.069, *P* < 0.01). However, in females the discrepancies in total taste bud numbers among stages were small and statistically insignificant (one-way ANOVA, F(5,21) = 1.42, *P* = 0.223). In males, taste bud number increased in young chicks and reached their peak at P3 (190 at P0 vs 260 at P3) and then returned to a relatively low level at later stages (P4–P8). Post-hoc Tukey HSD tests followed by one-way ANOVA showed that P3 male chickens had a significantly higher number of total taste buds than P4 (*P* < 0.05) and P8 (*P* < 0.01) chickens.

Taste buds in the base of oral cavity were frequently clustered around salivary gland openings (asterisks, Fig. 1F,I) in a rosette-like pattern. The size of bud clusters (number of taste buds per cluster) were analyzed (Fig. 4D–F) in the female-line males and male-line females. Similar to the number of total taste buds, the number of bud clusters (Fig. 4D) were also significantly higher in males than in females (two-way ANOVA, (F (5,49) = 3.08, *P* = 0.017). The differences between males and females were statistically significant at P0, P1, P3, and P5 stages (t- tests, *P* < 0.05, *P* < 0.05, *P* < 0.01, *P* < 0.05 respectively). Statistically significant changes over stages were observed in males (One-way ANOVA, F(5,28) = 6.057, *P* = 0.001) but not in females (One-way ANOVA, F(5,21) = 1.732, *P* = 0.171). The number of bud clusters in male chickens was greatest (60) at P3, which was significantly higher than at P0 (*P* < 0.05), P1 (*P* < 0.05), P4 (*P* < 0.05), and P8 (*P* < 0.01). The number of taste buds in each cluster in the base of the oral cavity of male and female chickens at different stages (P0–P8) was also analysed using Vimentin immunoreactivity (Fig. 4E,F). In males, the number of taste buds per cluster varied from 1–14 with 4 buds/cluster being the most prevalent (Fig. 4E). In contrast, females had a smaller range of taste buds per cluster, (1–9) and averaged 3 buds/cluster (Fig. 4F).

### Analysis of taste buds with scanning electron microscopy (SEM)

**Figure 5:**
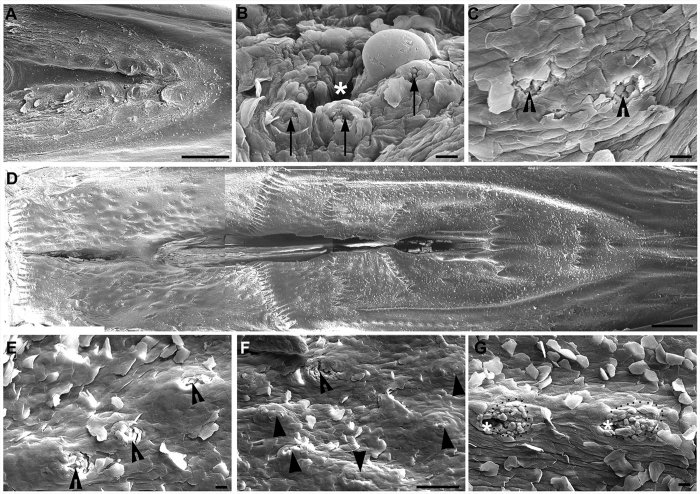
Scanning electron photomicrographs from the base of the oral cavity and palate in a P3 male chicken. (**A**) Low magnification of the base of the oral cavity showing the structure and topography of the oral surface. (**B)** A taste bud cluster showing 3 taste buds (arrows) located around a salivary gland opening (asterisks). (**C**) Representative images of taste buds (open arrowheads) that were not close to a salivary gland opening. (**D**) Low magnification image of the palate showing the topography of the oral surface. (**E,F**) Taste buds (open arrowheads) located in the absence of a salivary gland opening in the palate. Solid arrowheads in F point to tissue protrusions that were probably developing taste buds without an obvious taste pore. (**G**) Taste buds (dotted outlines) surrounding the salivary gland opening (asterisks) in the palate. Scale bars: 1 mm for (**A**) and (**D**); 20 μm for (**B,C**), (**E,G**); 100 μm for (**F**).

Taste buds labeled with Vimentin and α-Gustducin were significantly higher in number compared to those reported using SEM (~90 in the base of oral cavity, ~218 in the palate)^2^,^3^. To verify whether the differences were due to variations between strains of chickens, we used SEM to quantify the total number of taste pores from higher magnification images of the base of the oral cavity and the palate in the same strain of chickens (COBB 500, broiler-type, female-line males) at P3 (Fig. 5). The total number of readily identifiable taste pores was 74 (n = 2) in the base of the oral cavity and 185 (n = 2) in the palate, which was consistent with previous reports^5^,^12^.

In the base of the oral cavity (Fig. 5A), taste buds with pores (arrows, Fig. 5B) were identified surrounding salivary gland openings (asterisk, Fig. 5B). The salivary gland openings were deep with a diameter >20 μm, while the taste pore was shallow and small (<12 μm in diameter). The number of taste pores in a cluster varied from 1 to 5. Taste buds with a typical pore were also observed in the absence of a salivary gland opening (open arrowheads, Fig. 5C).

In the palate (Fig. 5D), the number of taste pores in a cluster was lower, ranging from 1 to 3. Compared to the base of the oral cavity, taste buds with a typical pore were more frequently observed in the absence of a salivary gland opening in the palate (open arrowheads, Fig. 5E,F). Protruded, taste bud-like structures without an obvious taste pore were also seen (arrowheads, Fig. 5F). Specified cell clusters (dotted outlines, Fig. 5G) were also seen surrounding salivary gland openings (asterisks, Fig. 5G), but individual taste pores were not obvious.

## Discussion

Chicken taste buds have previously been identified using SEM and histological analysis. However, molecular labeling has been limited to tissue sections. There has not been a method appropriate for visualizing all chicken taste buds in whole mount tissue using molecular markers. In the present study, we developed an efficient method that can be used to label chicken taste buds in oral epithelial sheets using traditional antibody labeling against Vimentin and α-Gustducin. With this new method, we identified a greater number of taste buds in oral tissue than previously reported. Furthermore, we were able to apply 2-photon microscopy to reveal ovoid- and tube-shaped taste buds in immunoreacted epithelial sheets.

In many organs and tissues, whole mount tissue can be used to evaluate phenotypes and changes during development. However, the gustatory epithelium has a strong permeability barrier that makes it difficult to label mature taste buds in whole tissue. Recently, we reported a protocol for peeling off adult murine tongue epithelial sheets following incubation with intralingually injected proteases and immediate fixation with 4% PFA. This enabled us to remove the permeability barrier in the basal region and reliably label taste buds in the tongue epithelium while maintaing the *in situ* shape^6^. In the present study, we adapted and optimized our protocol specifically for use in chicken oral tissue. We found that intact epithelial sheets can be obtained through sub-epithelially injected proteases, although a 1.5–2 hr incubation was required for chicken oral tissues compared to a 30-minute incubation for mouse tongue epithelium. This is probably due to the small sub-epithelial space and the unique structure of chicken oral tissue, which includes multiple thick and long spines.

Taste bud labeling using specific antibodies against Vimentin and α-Gustducin was successful in peeled epithelial sheets of the palate and the base of oral cavity where chicken taste buds are primarily located. The labeled taste buds were easy to identify under a light microscope, providing a highly efficient method for analyzing the distribution pattern and number of taste buds in the epithelial sheets. Moreover, the immunoreacted epithelial sheets were used for further examination of taste bud structure at the cellular level using laser-scanning confocal and 2-photon microscopy. 3-D reconstructed imaging analysis indicated that taste bud structure was different in different regions of the oral cavity. Chicken taste buds have been described as ovoid-shaped^3^. However, in the present study, both ovoid- and tube-shaped taste buds were observed in the palate. The method for obtaining peeled epithelial sheets developed in the present study will also be useful in studying gustatory and non-gustatory lingual epithelium with antibodies and probes for immunohistochemistry and *in situ* hybridization. Paired with traditional techniques, our method is efficient for phenotypical analysis of chicken gustatory tissue, and thus will facilitate studies on the development of taste buds and the role of taste buds in regulating feed intake of chickens. The protocol may be applicable to molecular labeling in other specialized epithelial appendages and in other species that include humans.

Our observations of taste buds using molecular markers bring forth new information about the sensory organs for taste in chickens. First, we found that previous reports underestimated the number of chicken taste buds. The number of taste buds in P3 female-line male chickens was ~507 in the palate and ~260 in the base of the oral cavity, which is much higher than the 218 (palate) and 91 (base of the oral cavity) taste buds previously counted with SEM^2^. The numbers of taste buds clustered in a rosette-like arrangement were up to 14 in female-line males and 9 in male-line females in the base of the oral cavity. These numbers were also higher than previously reported. Second, taste buds were distributed more broadly in the base of the oral cavity than previously reported, extending further to the lateral side of the lingual tissue. There has been a broad consensus that birds have lower taste acuity than mammals due to presence of fewer taste buds^13^,^14^. However, recent studies show that chickens have oral taste receptors sensitive to specific taste stimuli, e.g., bitter, umami, and fat via oral taste receptors^15^,^16^,^17^, suggesting that birds have a well-developed taste system^1^. Our findings regarding the higher number of taste buds in chickens indicate a potentially larger than previously appreciated impact of taste on feeding behaviors in birds.

It has been reported that the number of taste buds in the oral cavity measured at P5 varies among different breeds of chicken, such as in White Leghorn layer-type males, Rhode Island Red layer-type males, and broiler-type males^4^. In the present study, we found that the number of taste buds and clusters formed by taste buds varied by gender even in the same breed: female-line males continued to have more taste buds and clusters than females and male-line males soon after hatch. We also found that the number of taste buds in the cluster was higher in female-line males than in females: one cluster contained 4 taste buds in males and 3 taste buds in females.

Another interesting difference was that in female-line male chickens, the numbers of taste buds and clusters change with age from P0–P8. We found that taste bud number in males increases after hatch, peaks at P3, and then deceases at later stages. In contrast, in female and male-line male chickens the numbers of taste buds in the base of oral cavity were more stable during post-hatch stages. In previous studies analyzing development of taste buds at P0, P50–60, and adult, no differences were found among these stages in Anak strain broiler chicks^2^,^5^. Our finding of continuing development of taste buds in female-line male chickens after P0 provides a time window when taste bud formation and therefore taste sensing can be modified in early hatched chickens.

Although the mechanisms underlying the different development of taste buds in males and females are not clear, there are many possible candidates for future study, including genetic background, sex hormones, and growth factors. A significant difference between female-line males and male-line females and females from both lines was observed from P0, which makes it reasonable to speculate that the difference in number of taste buds exists during the initial development of taste buds in the late embryo. The number of taste buds is an important factor that determines taste sensitivity^4^ and is associated with feeding behavior. Further studies on how taste bud formation is regulated will be beneficial for improving chickens’ healthy feed choices and intake, thus improving animal health and productivity.

## Materials and Methods

### Animal and tissue collection

The use of animals throughout the study was approved by The University of Georgia Institutional Animal Care and Use Committee and was in compliance with the National Institutes of Health Guidelines for the care and use of animals in research.

Newly hatched Cobb 500 (P0) broiler-type chickens, both genders from male- and female-lines, were received from the Cobb-Vantress., Inc, Cleveland Hatchery, Georgia. The chicks were housed in separate cages in an animal facility at the Department of Animal Science, University of Georgia, until P8 (8 days). The brooder temperature was ~35 °C and room temperature was maintained at 30 °C with food (starter feed) and water available *ad libitum* under a 12–12 hr light-dark cycle.

P0, P1, P3, P4, P5, and P8 chicks were euthanized by decapitation. The oral tissue in the palate, base of the oral cavity, and posterior region of the tongue were dissected and processed for different analyses as below.

### Oral epithelial sheet peeling

The palate and base of the oral cavity were dissected and briefly rinsed in 0.1 M PBS. An enzyme mixture of Collagenase A (1 mg/ml, Cat# 10103578001, Roche Diagnostics) and Dispase II (2.5 mg/ml, Cat# 04942078001, Roche Diagnostics) was injected (6 ml in total) into the sub-epithelial space of the palate, the base of the oral cavity, and the posterior region of the tongue, followed by incubation at 37 °C for 2 hr. Following enzymatic tissue digestion, the tissue was immediately fixed in 4% paraformaldehyde (PFA) for 1 hr at room temperature followed by brief rinse in 0.1 M phosphate buffered saline (PBS). The soft tissue regions containing taste buds were dissected from the beaks, and the epithelial sheets of the palate, base of the oral cavity, and posterior tongue were peeled off from the underlying connective tissue. After thorough rinsing in 0.1 M PBS, the epithelial sheets were processed for immunohistochemistry.

### Vimentin and α-Gustducin immunohistochemistry in epithelial sheets

Peeled epithelial sheets were evaluated under a stereomicroscope. Intact sheets from chicks at different stages (P0, P1, P3, P4, P5, P8) were selected (n = 3–7 for each stage and gender) for further processing. Epithelial sheets were rinsed in 0.1 M PBS. Non-specific staining was blocked with 10% normal donkey serum (NDS) (Cat# D9663-10 ml, Sigma-Aldrich) and 10% Bovine Serum Albumin Fraction V (BSA) (Cat#15260-037, ThermoFisher Scientific) in 0.1 M PBS containing 0.3% Triton-X100 (PBS-X) overnight at 4 °C. Sheets were then incubated with primary antibodies against Vimentin (1:200, abcam 28028; Vim3B4, mouse monoclonal antibody, Abcam, Cambridge, MA) and α-Gustducin (1:250, serum of rabbit immunized with chicken α-Gustducin, generated by Dr. Shoji Tabata Lab)^9^ in 1% NDS, 1% BSA in PBS-X for 72 hr at 4 °C. Following rinses in 0.1 M PBS, epithelial sheets were incubated with Alexa Fluor 488 conjugated donkey anti-mouse (1:500, Code: 715-545-150, Jackson Immuno Research Laboratories, Inc) and Alexa Fluor 647 conjugated donkey anti-rabbit secondary antibody (1:500, Code: 711-605-152; Jackson Immuno Research Laboratories, Inc) in 1% NDS in PBS-X overnight at 4 °C. The epithelial sheets were rinsed in 0.1 M PBS and photomicrographed using a SZX2-ILLT Olympus stereomicroscope with CellSens software (Olympus, Life sciences). Z-projection confocal images were taken using a LSM710 laser-scanning confocal microscope using ZEN 2012 software in the Biomedical Microscopy Core (BMC) at the University of Georgia for analysis of labeled taste buds at the cellular level.

### 2-photon microscopy and 3-D image reconstruction of chicken taste buds

Epithelial sheets from the base of the oral cavity and the palate of P3 chickens immunoreacted against Vimentin and α-Gustducin used for 3-D imaging. The 3-D images were acquired with a home-built 2-photon microscope. A 1550 nm, 370 femtosecond pulsed fiber laser (Calmar Cazadero) with a wavelength of 1550 nm and repetition rate of 10 MHz was used. The beam was frequency doubled using a second harmonic generation (SHG) crystal (Newlight photonics) to produce a 775 nm beam that was used for 2-photon excitation of the sample. The beam power was modulated using a Pockels cell (Conoptics) and the beam was scanned over the sample by a resonant-galvanometer (fast axis – slow axis) scanner (Sutter instruments RESSCAN-GEN). A 60x Olympus (LUMFLN60x) water immersion objective with NA of 1.1 was used for imaging. Z-scanning was performed using an X-Y-Z stage from Sutter Instruments (MPC-200). Signal emitted from the sample was separated into two channels. 690/40 nm and 520/50 nm filters were used for collection of signal from Alexa Fluor 647 and Alexa Fluor 488, respectively. Photon multiplier tubes (PMT) from Hamamatsu were used for collection of the signal. A transimpedance amplifier (Edmund Optics 59–178) was used for each channel to convert current output of PMTs to an amplified voltage. National Instruments DAQ cards and FPGA modules were used for control and synchronization of the system and digitization of the detected signal. The Matlab-based open-source software ScanImage^18^ was employed to control the microscope. More information on the laser and optical setup can be found in recent reports^19^.

### Scanning electron microscopy (SEM)

The palate and base of the oral cavity of P3 male-line female chickens were dissected and fixed in SEM fixative containing 4% PFA, 2.5% glutaraldehyde in 0.1 M PBS over 48 hr at room temperature. The tissue was trimmed and thoroughly rinsed in 0.1 M PBS and processed further into a series of 1% osmium tetroxide (OsO_4_), 1% tannic acid and 1% OsO_4_ aqueous solutions for 1 hr on ice. The tissues were dehydrated sequentially with ethanol (35%, 50%, 70%, 95% and 100%; three times at each concentration for 2 hr each). Specimens were dried completely using a critical point dryer (Autosamdri-814 Critical Point Dryer, Tousimis Research Corporation, Rockville, MD, USA). The samples were mounted onto SEM stub, sputter coated with gold and photomicrographs were taken using a Zeiss 1450EP scanning electron microscope (Carl Zeiss MicroImaging, Inc., NY, and Oxford Instruments X-Ray Technology, Inc., CA).

### Quantification and statistical analysis

Quantitative analysis was performed for the total number of taste buds, taste bud clusters, and taste bud number per cluster in the base of the oral cavity in both genders from both male- and female-lines at different stages (P0, P1, P3, P4, P5, and P8 (n = 3–7). The total number of taste buds in the palate of the female-line males and male-line females at selected stages (P1 and P3, n = 3–5). Vimentin and/or α-Gustducin immunosignals were used to visualize taste buds in the oral epithelial sheets. All quantification was carried out manually by the same investigator for consistency among groups using photomicrographs obtained from an Olympus stereomicroscope. The quantification of taste bud numbers in the base of the oral cavity of the male-line and female-line chickens at P1 and P3 (Fig. 4A) was double-blinded to avoid bias. The quantification data are represented as means ± standard deviation (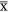 ± SD; n = 3–7). One-way or Two-way analysis of variance (ANOVA) followed by a t-test or Post Hoc Tukey HSD Tests was performed to test the statistical significance of differences between males and females, and between the examined stages. A *P* value < 0.05 was taken as a statistically significant difference.

## Additional Information

**How to cite this article**: Rajapaksha, P. *et al.* Labeling and analysis of chicken taste buds using molecular markers in oral epithelial sheets. *Sci. Rep.* **6**, 37247; doi: 10.1038/srep37247 (2016).

**Publisher’s note**: Springer Nature remains neutral with regard to jurisdictional claims in published maps and institutional affiliations.
